# Ethyl 1-(4-chloro­phen­yl)-3-[1-(4-meth­oxy­phen­yl)-4-oxo-3-phenyl­azetidin-2-yl]-2-nitro-2,3,10,10a-tetra­hydro-1*H*,5*H*-pyr­rolo[1,2-*b*]isoquinoline-10a-carboxyl­ate

**DOI:** 10.1107/S1600536808030961

**Published:** 2008-09-30

**Authors:** S. Sundaresan, P. Ramesh, N. Arumugam, R. Raghunathan, M. N. Ponnuswamy

**Affiliations:** a Centre of Advanced Study in Crystallography and Biophysics, University of Madras, Guindy Campus, Chennai 600025, India; b Department of Physics, Presidency College (Autonomous), Chennai 600005, India; c Department of Organic Chemistry, University of Madras, Guindy Campus, Chennai 600025, India

## Abstract

In the title compound, C_37_H_34_ClN_3_O_6_, the pyrrolidine and piperidine rings adopt envelope and boat conformations, respectively. The β-lactam ring is planar and forms dihedral angles of 21.3 (2) and 73.9 (2)°, respectively, with the attached methoxy­phenyl and phenyl rings. Intra­molecular C—H⋯O and C—H⋯N hydrogen bonds are observed. Centrosym­metrically related mol­ecules are linked together by weak C—H⋯O hydrogen bonds to form dimers.

## Related literature

For the biological properties of β-lactam derivatives, see: Borthwick *et al.* (1998 ▶); Brakhage (1998 ▶); Burnett (1994 ▶); Han *et al.* (1995 ▶); Vaccaro & Davis (1998 ▶); Vaccaro *et al.* (1998 ▶). For puckering and asymmetry parameters, see: Cremer & Pople (1975 ▶); Nardelli (1983 ▶).
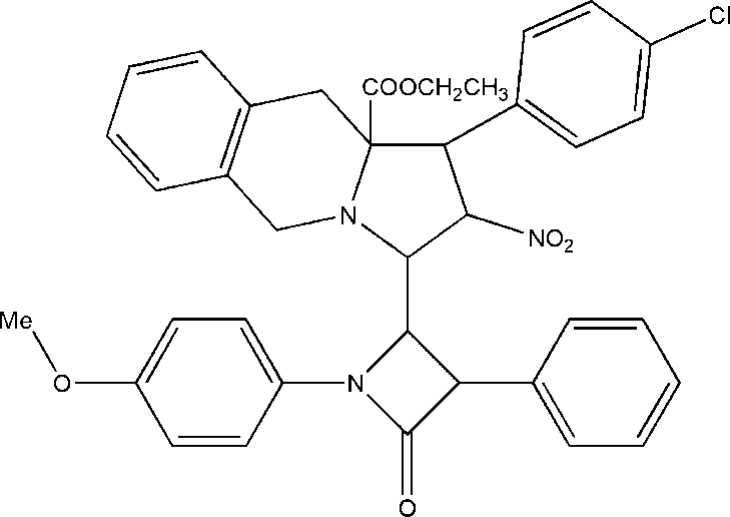


## Experimental

### 

#### Crystal data

C_37_H_34_ClN_3_O_6_
*M* *_r_* = 652.12
Monoclinic, 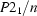

*a* = 9.1723 (2) Å
*b* = 18.0452 (4) Å
*c* = 19.7475 (5) Å
β = 100.638 (1)°
*V* = 3212.35 (13) Å^3^
*Z* = 4
Mo *K*α radiation
μ = 0.17 mm^−1^
*T* = 293 (2) K
0.22 × 0.20 × 0.17 mm

#### Data collection

Bruker kappa APEXII area-detector diffractometer
Absorption correction: multi-scan (*SADABS*; Sheldrick, 2001 ▶) *T*_min_ = 0.963, *T*_max_ = 0.971
30325 measured reflections
5582 independent reflections
3760 reflections with *I* > 2σ(*I*)
*R*_int_ = 0.034

#### Refinement

*R*[*F* ^2^ > 2σ(*F* ^2^)] = 0.047
*wR*(*F* ^2^) = 0.135
*S* = 1.02
5582 reflections
426 parameters
H-atom parameters constrained
Δρ_max_ = 0.34 e Å^−3^
Δρ_min_ = −0.27 e Å^−3^

### 

Data collection: *APEX2* (Bruker, 2004 ▶); cell refinement: *SAINT* (Bruker, 2004 ▶); data reduction: *SAINT*; program(s) used to solve structure: *SHELXS97* (Sheldrick, 2008 ▶); program(s) used to refine structure: *SHELXL97* (Sheldrick, 2008 ▶); molecular graphics: *ORTEP-3* (Farrugia, 1997 ▶); software used to prepare material for publication: *SHELXL97* and *PLATON* (Spek, 2003 ▶).

## Supplementary Material

Crystal structure: contains datablocks global, I. DOI: 10.1107/S1600536808030961/ci2676sup1.cif

Structure factors: contains datablocks I. DOI: 10.1107/S1600536808030961/ci2676Isup2.hkl

Additional supplementary materials:  crystallographic information; 3D view; checkCIF report

## Figures and Tables

**Table 1:** Hydrogen-bond geometry (Å, °)

*D*—H⋯*A*	*D*—H	H⋯*A*	*D*⋯*A*	*D*—H⋯*A*
C2—H2*A*⋯N27	0.97	2.53	3.196 (3)	126
C24—H24⋯O3	0.98	2.58	3.186 (3)	121
C25—H25⋯O6^i^	0.98	2.60	3.479 (3)	150
C35—H35⋯O5	0.93	2.60	3.157 (3)	119
C40—H40*A*⋯O4^i^	0.96	2.49	3.232 (4)	134

Symmetry code: (i) 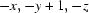.

**Fig. 1.**
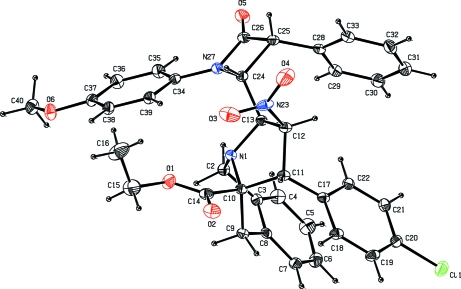
The molecular structure of the title compound, showing 20% probability displacement ellipsoids and the atom-numbering scheme.

## supplementary crystallographic information

### Comment

β-Lactams have been shown to exhibit high antibacterial activity. 1,3,4-Trisubstituted β-lactams are potent cholesterol absorption inhibitors (Vaccaro & Davis, 1998; Vaccaro *et al.*, 1998; Burnett, 1994), human cylomegalonims and protease inhibitors (Borthwick *et al.*, 1998) and thrombin inhibitors (Han *et al.*, 1995). The most commonly used β-lactam antibiotics for the therapy of infectious diseases are penicillin and cephalosporins (Brakhage, 1998). In view of these importance, the crystal structure determination of the title compound was carried out.

The pyrrolidine and piperidine rings in the molecule adopt envelope and boat conformations, respectively. The puckering parameters (Cremer & Pople, 1975) and the asymmetry parameter (Nardelli, 1983) for the pyrrolidine ring are q_2_ = 0.292 (3) Å, π = 99.6 (5)° and Δ_S_(C12) = 4.4 (3)°, and for the piperidine ring q_2_ = 0.630 (3) Å, q_3_ = 0.004 (3) Å, π = 61.2 (2)° and Δ_S_(C2) = Δ_S_(C9) = 1.2 (2)°. The ethylcarboxylate group adopts an extended conformation. The sum of angles at atom N27 of the β-lactam ring system [356.9°] is in accordance with *sp*^2^ hybridization. The β-lactam ring is planar and the keto O5 atom deviates from this plane by -0.005 (2) Å. The methoxyphenyl (C34—C39) and (C28—C33) phenyl rings form dihedral angles of 21.3 (2) and 73.9 (2)°, respectively, with the β-lactam ring. C—H···O and C—H···N types of intramolecular hydrogen bonds are observed.

In the crystal structure, atoms C25 and C40 of the molecule at (*x*, *y*, *z*) donate one proton each to atoms O6 and O4 of the molecule at (- *x*, 1 - *y*, -*z*), forming a cyclic centrosymmetric dimer.

### Experimental

To a stirred solution of 5-[1'-*N*-(*p*-methoxyphenyl-azetidine-2'-one]-4-nitro-3-(*p*-chloro)-phenyl-2-ethoxycarbonyl-2-benzyl-pyrrlolidine (1 mmol) in dry chloroform (20 ml) *para* formaldehyde (1 mmol) and then trifluroacetic acid (0.1 mmol) were added at room temperature. After completion of the reaction, the mixture was washed with water and dried over. The solvent was removed under the reduced pressure and the crude product was subjected to column chromatography with hexane-ethyl acetate (9:1) to obtain pure cyclized product. The compound was recrystallized from ethyl acetate

### Refinement

All H atoms were positioned geometrically (C—H = 0.93–0.98 Å) and allowed to ride on their parent atoms, with *U*_iso_(H) = 1.5*U*_eq_(C) for methyl H atoms and 1.2*U*_eq_(C) for other H atoms. Large thermal vibrations of atoms C15 and C16 resulted in the shortening of C15—C16 length.

### Crystal data

**Table:**

C_37_H_34_ClN_3_O_6_	*F*(000) = 1368
*M**_r_* = 652.12	*D*_x_ = 1.348 Mg m^−^^3^
Monoclinic, *P*2_1_/*n*	Mo *K*α radiation, λ = 0.71073 Å
Hall symbol: -P 2yn	Cell parameters from 5002 reflections
*a* = 9.1723 (2) Å	θ = 1.5–24.9°
*b* = 18.0452 (4) Å	µ = 0.17 mm^−^^1^
*c* = 19.7475 (5) Å	*T* = 293 K
β = 100.638 (1)°	Block, colourless
*V* = 3212.35 (13) Å^3^	0.22 × 0.20 × 0.17 mm
*Z* = 4	

### Data collection

**Table:**

Bruker kappa APEXII area-detector diffractometer	5582 independent reflections
Radiation source: fine-focus sealed tube	3760 reflections with *I* > 2σ(*I*)
graphite	*R*_int_ = 0.034
ω and φ scans	θ_max_ = 24.9°, θ_min_ = 1.5°
Absorption correction: multi-scan (SADABS; Sheldrick, 2001)	*h* = −10→10
*T*_min_ = 0.963, *T*_max_ = 0.971	*k* = −20→21
30325 measured reflections	*l* = −23→23

### Refinement

**Table:**

Refinement on *F*^2^	Primary atom site location: structure-invariant direct methods
Least-squares matrix: full	Secondary atom site location: difference Fourier map
*R*[*F*^2^ > 2σ(*F*^2^)] = 0.047	Hydrogen site location: inferred from neighbouring sites
*wR*(*F*^2^) = 0.135	H-atom parameters constrained
*S* = 1.03	*w* = 1/[σ^2^(*F*_o_^2^) + (0.0537*P*)^2^ + 1.5127*P*] where *P* = (*F*_o_^2^ + 2*F*_c_^2^)/3
5582 reflections	(Δ/σ)_max_ = 0.001
426 parameters	Δρ_max_ = 0.34 e Å^−^^3^
0 restraints	Δρ_min_ = −0.27 e Å^−^^3^

### Special details

**Table:**

Geometry. All e.s.d.'s (except the e.s.d. in the dihedral angle between two l.s. planes) are estimated using the full covariance matrix. The cell e.s.d.'s are taken into account individually in the estimation of e.s.d.'s in distances, angles and torsion angles; correlations between e.s.d.'s in cell parameters are only used when they are defined by crystal symmetry. An approximate (isotropic) treatment of cell e.s.d.'s is used for estimating e.s.d.'s involving l.s. planes.
Refinement. Refinement of *F*^2^ against ALL reflections. The weighted *R*-factor *wR* and goodness of fit *S* are based on *F*^2^, conventional *R*-factors *R* are based on *F*, with *F* set to zero for negative *F*^2^. The threshold expression of *F*^2^ > σ(*F*^2^) is used only for calculating *R*-factors(gt) *etc*. and is not relevant to the choice of reflections for refinement. *R*-factors based on *F*^2^ are statistically about twice as large as those based on *F*, and *R*- factors based on ALL data will be even larger.

### Fractional atomic coordinates and isotropic or equivalent isotropic displacement parameters (Å^2^)

**Table:**

	*x*	*y*	*z*	*U*_iso_*/*U*_eq_	
Cl1	0.26960 (11)	0.43479 (5)	0.60642 (4)	0.0979 (3)	
O1	−0.1144 (2)	0.31964 (12)	0.15686 (11)	0.0870 (6)	
O2	−0.2380 (2)	0.31467 (12)	0.24445 (12)	0.0914 (7)	
O3	−0.2459 (3)	0.47487 (16)	0.18822 (18)	0.1215 (11)	
O4	−0.2416 (4)	0.58070 (18)	0.23474 (14)	0.1523 (14)	
O5	0.3329 (2)	0.63293 (11)	0.11354 (11)	0.0839 (6)	
O6	0.2021 (2)	0.31726 (12)	−0.07279 (10)	0.0823 (6)	
N1	0.08498 (19)	0.41518 (10)	0.21858 (10)	0.0468 (5)	
C2	0.2354 (3)	0.38685 (14)	0.21800 (13)	0.0556 (6)	
H2A	0.2891	0.4229	0.1957	0.067*	
H2B	0.2280	0.3416	0.1911	0.067*	
C3	0.3207 (3)	0.37143 (13)	0.28843 (13)	0.0537 (6)	
C4	0.4641 (3)	0.39488 (18)	0.31274 (17)	0.0771 (8)	
H4	0.5137	0.4230	0.2847	0.093*	
C5	0.5336 (3)	0.3767 (2)	0.3782 (2)	0.0940 (10)	
H5	0.6295	0.3934	0.3947	0.113*	
C6	0.4625 (4)	0.33415 (19)	0.41941 (17)	0.0836 (9)	
H6	0.5113	0.3209	0.4633	0.100*	
C7	0.3198 (3)	0.31095 (14)	0.39631 (14)	0.0644 (7)	
H7	0.2714	0.2824	0.4246	0.077*	
C8	0.2481 (3)	0.33003 (12)	0.33104 (13)	0.0499 (6)	
C9	0.0928 (3)	0.30842 (12)	0.30105 (13)	0.0547 (6)	
H9A	0.0441	0.2909	0.3377	0.066*	
H9B	0.0945	0.2678	0.2690	0.066*	
C10	0.0017 (2)	0.37342 (12)	0.26295 (12)	0.0487 (6)	
C11	−0.0500 (3)	0.43205 (12)	0.31170 (13)	0.0513 (6)	
H11	−0.1545	0.4222	0.3132	0.062*	
C12	−0.0423 (3)	0.50536 (13)	0.27378 (13)	0.0551 (6)	
H12	−0.0162	0.5457	0.3070	0.066*	
C13	0.0807 (3)	0.49450 (12)	0.23229 (12)	0.0479 (5)	
H13	0.1747	0.5079	0.2621	0.058*	
C14	−0.1333 (3)	0.33509 (15)	0.22050 (16)	0.0654 (7)	
C15	−0.2295 (5)	0.2774 (2)	0.1122 (2)	0.1293 (16)	
H15A	−0.1831	0.2395	0.0886	0.155*	
H15B	−0.2911	0.2528	0.1403	0.155*	
C16	−0.3162 (5)	0.3200 (3)	0.0650 (2)	0.163 (2)	
H16A	−0.3704	0.3544	0.0880	0.245*	
H16B	−0.3846	0.2891	0.0348	0.245*	
H16C	−0.2554	0.3467	0.0387	0.245*	
C17	0.0339 (3)	0.43451 (12)	0.38492 (13)	0.0522 (6)	
C18	−0.0165 (3)	0.39231 (14)	0.43459 (14)	0.0624 (7)	
H18	−0.1011	0.3634	0.4220	0.075*	
C19	0.0560 (3)	0.39233 (16)	0.50219 (15)	0.0717 (8)	
H19	0.0207	0.3635	0.5348	0.086*	
C20	0.1801 (3)	0.43493 (16)	0.52118 (14)	0.0677 (8)	
C21	0.2316 (3)	0.47828 (15)	0.47366 (15)	0.0696 (7)	
H21	0.3153	0.5077	0.4869	0.083*	
C22	0.1586 (3)	0.47789 (14)	0.40612 (13)	0.0610 (7)	
H22	0.1938	0.5074	0.3740	0.073*	
N23	−0.1895 (3)	0.52072 (17)	0.22867 (14)	0.0772 (8)	
C24	0.0636 (3)	0.54308 (12)	0.16801 (12)	0.0512 (6)	
H24	−0.0372	0.5412	0.1409	0.061*	
C25	0.1223 (3)	0.62490 (13)	0.18009 (13)	0.0559 (6)	
H25	0.0496	0.6593	0.1546	0.067*	
C26	0.2339 (3)	0.60255 (14)	0.13526 (13)	0.0611 (7)	
N27	0.1771 (2)	0.53255 (11)	0.12491 (10)	0.0555 (5)	
C28	0.1739 (3)	0.65324 (12)	0.25172 (13)	0.0547 (6)	
C29	0.3113 (3)	0.63541 (14)	0.28979 (16)	0.0678 (7)	
H29	0.3770	0.6073	0.2698	0.081*	
C30	0.3525 (4)	0.65882 (17)	0.35720 (18)	0.0828 (9)	
H30	0.4448	0.6456	0.3823	0.099*	
C31	0.2596 (4)	0.70088 (18)	0.38705 (18)	0.0861 (9)	
H31	0.2877	0.7163	0.4325	0.103*	
C32	0.1253 (4)	0.72025 (16)	0.35001 (18)	0.0813 (9)	
H32	0.0616	0.7494	0.3703	0.098*	
C33	0.0822 (3)	0.69735 (14)	0.28273 (16)	0.0665 (7)	
H33	−0.0097	0.7118	0.2579	0.080*	
C34	0.1861 (3)	0.47852 (13)	0.07403 (12)	0.0533 (6)	
C35	0.3076 (3)	0.47553 (16)	0.04257 (15)	0.0700 (8)	
H35	0.3843	0.5094	0.0547	0.084*	
C36	0.3168 (3)	0.42258 (16)	−0.00697 (15)	0.0725 (8)	
H36	0.3991	0.4213	−0.0283	0.087*	
C37	0.2053 (3)	0.37216 (15)	−0.02476 (13)	0.0596 (6)	
C38	0.0838 (3)	0.37514 (15)	0.00705 (13)	0.0612 (7)	
H38	0.0073	0.3411	−0.0050	0.073*	
C39	0.0741 (3)	0.42744 (14)	0.05597 (13)	0.0573 (6)	
H39	−0.0084	0.4286	0.0772	0.069*	
C40	0.3268 (3)	0.30843 (19)	−0.10496 (16)	0.0824 (9)	
H40A	0.3477	0.3545	−0.1256	0.124*	
H40B	0.3062	0.2710	−0.1399	0.124*	
H40C	0.4112	0.2939	−0.0712	0.124*	

### Atomic displacement parameters (Å^2^)

**Table:**

	*U*^11^	*U*^22^	*U*^33^	*U*^12^	*U*^13^	*U*^23^
Cl1	0.1261 (7)	0.1070 (6)	0.0593 (5)	0.0466 (5)	0.0135 (5)	−0.0010 (4)
O1	0.0823 (14)	0.1021 (16)	0.0680 (14)	−0.0362 (12)	−0.0090 (11)	0.0012 (11)
O2	0.0641 (13)	0.0896 (15)	0.1228 (19)	−0.0263 (11)	0.0235 (12)	0.0104 (13)
O3	0.0639 (15)	0.0989 (19)	0.183 (3)	−0.0030 (13)	−0.0253 (17)	0.0309 (19)
O4	0.183 (3)	0.162 (3)	0.107 (2)	0.128 (2)	0.0138 (19)	0.0210 (18)
O5	0.0955 (15)	0.0691 (12)	0.0985 (16)	−0.0293 (11)	0.0479 (13)	−0.0002 (11)
O6	0.0805 (13)	0.0942 (15)	0.0781 (14)	−0.0059 (11)	0.0301 (11)	−0.0264 (11)
N1	0.0446 (11)	0.0440 (10)	0.0542 (12)	0.0015 (8)	0.0153 (9)	0.0028 (8)
C2	0.0516 (14)	0.0588 (14)	0.0607 (16)	0.0085 (11)	0.0215 (12)	−0.0004 (12)
C3	0.0449 (14)	0.0546 (14)	0.0628 (16)	0.0097 (11)	0.0132 (12)	−0.0047 (12)
C4	0.0477 (16)	0.090 (2)	0.096 (2)	0.0049 (14)	0.0180 (16)	0.0048 (17)
C5	0.0507 (18)	0.113 (3)	0.109 (3)	0.0087 (18)	−0.0078 (19)	−0.001 (2)
C6	0.075 (2)	0.094 (2)	0.073 (2)	0.0268 (18)	−0.0099 (17)	−0.0029 (18)
C7	0.0673 (18)	0.0575 (15)	0.0676 (18)	0.0192 (13)	0.0107 (14)	0.0045 (13)
C8	0.0523 (14)	0.0401 (12)	0.0574 (15)	0.0105 (10)	0.0102 (12)	−0.0025 (11)
C9	0.0590 (15)	0.0438 (13)	0.0624 (16)	0.0010 (11)	0.0135 (12)	0.0045 (11)
C10	0.0460 (13)	0.0449 (12)	0.0570 (15)	−0.0022 (10)	0.0147 (11)	0.0061 (10)
C11	0.0448 (13)	0.0510 (13)	0.0624 (16)	0.0063 (10)	0.0210 (12)	0.0128 (11)
C12	0.0566 (15)	0.0506 (13)	0.0628 (16)	0.0099 (11)	0.0235 (12)	0.0121 (11)
C13	0.0506 (13)	0.0434 (12)	0.0524 (14)	0.0012 (10)	0.0165 (11)	0.0047 (10)
C14	0.0532 (16)	0.0605 (16)	0.080 (2)	−0.0077 (12)	0.0061 (14)	0.0148 (14)
C15	0.116 (3)	0.118 (3)	0.126 (3)	−0.056 (3)	−0.049 (3)	0.015 (3)
C16	0.147 (4)	0.197 (5)	0.117 (4)	−0.089 (4)	−0.054 (3)	0.056 (4)
C17	0.0563 (15)	0.0456 (13)	0.0607 (16)	0.0136 (11)	0.0268 (12)	0.0062 (11)
C18	0.0636 (16)	0.0617 (16)	0.0685 (18)	0.0128 (12)	0.0291 (14)	0.0159 (13)
C19	0.085 (2)	0.0726 (18)	0.0664 (19)	0.0274 (16)	0.0365 (17)	0.0206 (15)
C20	0.084 (2)	0.0662 (17)	0.0564 (17)	0.0298 (16)	0.0217 (15)	0.0001 (14)
C21	0.081 (2)	0.0640 (17)	0.0662 (19)	0.0074 (14)	0.0208 (16)	−0.0104 (14)
C22	0.0775 (18)	0.0541 (15)	0.0570 (17)	0.0032 (13)	0.0275 (14)	−0.0006 (12)
N23	0.0689 (17)	0.0873 (19)	0.0842 (19)	0.0343 (15)	0.0372 (15)	0.0408 (15)
C24	0.0510 (14)	0.0512 (13)	0.0535 (14)	−0.0018 (10)	0.0155 (11)	0.0061 (11)
C25	0.0631 (16)	0.0464 (13)	0.0597 (16)	−0.0005 (11)	0.0150 (13)	0.0100 (11)
C26	0.0712 (17)	0.0539 (15)	0.0612 (16)	−0.0088 (13)	0.0204 (14)	0.0096 (12)
N27	0.0630 (13)	0.0520 (12)	0.0564 (13)	−0.0118 (9)	0.0240 (10)	0.0015 (9)
C28	0.0599 (16)	0.0421 (13)	0.0632 (16)	−0.0049 (11)	0.0139 (13)	0.0076 (11)
C29	0.0655 (18)	0.0538 (15)	0.084 (2)	−0.0015 (13)	0.0131 (16)	0.0007 (14)
C30	0.078 (2)	0.0727 (19)	0.089 (2)	−0.0049 (16)	−0.0078 (18)	−0.0006 (17)
C31	0.103 (3)	0.075 (2)	0.077 (2)	−0.0136 (19)	0.006 (2)	−0.0069 (17)
C32	0.098 (2)	0.0653 (18)	0.089 (2)	−0.0054 (17)	0.037 (2)	−0.0132 (16)
C33	0.0673 (17)	0.0526 (15)	0.082 (2)	0.0022 (13)	0.0190 (15)	0.0036 (14)
C34	0.0600 (15)	0.0546 (14)	0.0485 (14)	−0.0056 (11)	0.0181 (12)	0.0076 (11)
C35	0.0649 (17)	0.0724 (18)	0.079 (2)	−0.0210 (14)	0.0286 (15)	−0.0059 (15)
C36	0.0656 (18)	0.082 (2)	0.079 (2)	−0.0086 (15)	0.0366 (15)	−0.0050 (16)
C37	0.0623 (16)	0.0672 (16)	0.0516 (15)	−0.0007 (13)	0.0166 (13)	−0.0013 (12)
C38	0.0623 (16)	0.0690 (16)	0.0545 (15)	−0.0145 (13)	0.0163 (13)	−0.0038 (13)
C39	0.0551 (15)	0.0685 (16)	0.0522 (15)	−0.0111 (12)	0.0203 (12)	0.0007 (12)
C40	0.079 (2)	0.099 (2)	0.0722 (19)	0.0267 (17)	0.0224 (16)	−0.0084 (17)

### Geometric parameters (Å, °)

**Table:**

Cl1—C20	1.731 (3)	C16—H16C	0.96
O1—C14	1.329 (3)	C17—C22	1.385 (4)
O1—C15	1.460 (4)	C17—C18	1.387 (3)
O2—C14	1.204 (3)	C18—C19	1.377 (4)
O3—N23	1.199 (4)	C18—H18	0.93
O4—N23	1.198 (3)	C19—C20	1.368 (4)
O5—C26	1.205 (3)	C19—H19	0.93
O6—C37	1.368 (3)	C20—C21	1.371 (4)
O6—C40	1.416 (3)	C21—C22	1.378 (4)
N1—C13	1.459 (3)	C21—H21	0.93
N1—C10	1.472 (3)	C22—H22	0.93
N1—C2	1.473 (3)	C24—N27	1.474 (3)
C2—C3	1.491 (3)	C24—C25	1.575 (3)
C2—H2A	0.97	C24—H24	0.98
C2—H2B	0.97	C25—C28	1.496 (3)
C3—C4	1.381 (4)	C25—C26	1.526 (4)
C3—C8	1.384 (3)	C25—H25	0.98
C4—C5	1.372 (4)	C26—N27	1.367 (3)
C4—H4	0.93	N27—C34	1.413 (3)
C5—C6	1.368 (5)	C28—C33	1.381 (4)
C5—H5	0.93	C28—C29	1.381 (4)
C6—C7	1.370 (4)	C29—C30	1.381 (4)
C6—H6	0.93	C29—H29	0.93
C7—C8	1.378 (3)	C30—C31	1.354 (5)
C7—H7	0.93	C30—H30	0.93
C8—C9	1.490 (3)	C31—C32	1.357 (4)
C9—C10	1.552 (3)	C31—H31	0.93
C9—H9A	0.97	C32—C33	1.378 (4)
C9—H9B	0.97	C32—H32	0.93
C10—C14	1.527 (4)	C33—H33	0.93
C10—C11	1.562 (3)	C34—C35	1.374 (3)
C11—C17	1.508 (3)	C34—C39	1.377 (3)
C11—C12	1.528 (3)	C35—C36	1.381 (4)
C11—H11	0.98	C35—H35	0.93
C12—N23	1.500 (4)	C36—C37	1.365 (4)
C12—C13	1.524 (3)	C36—H36	0.93
C12—H12	0.98	C37—C38	1.378 (3)
C13—C24	1.527 (3)	C38—C39	1.365 (3)
C13—H13	0.98	C38—H38	0.93
C15—C16	1.347 (5)	C39—H39	0.93
C15—H15A	0.97	C40—H40A	0.96
C15—H15B	0.97	C40—H40B	0.96
C16—H16A	0.96	C40—H40C	0.96
C16—H16B	0.96		
			
C14—O1—C15	118.3 (3)	C19—C18—H18	119.3
C37—O6—C40	118.5 (2)	C17—C18—H18	119.3
C13—N1—C10	110.93 (17)	C20—C19—C18	119.6 (3)
C13—N1—C2	113.53 (18)	C20—C19—H19	120.2
C10—N1—C2	114.62 (17)	C18—C19—H19	120.2
N1—C2—C3	112.88 (19)	C19—C20—C21	120.5 (3)
N1—C2—H2A	109.0	C19—C20—Cl1	119.1 (2)
C3—C2—H2A	109.0	C21—C20—Cl1	120.3 (3)
N1—C2—H2B	109.0	C20—C21—C22	119.5 (3)
C3—C2—H2B	109.0	C20—C21—H21	120.3
H2A—C2—H2B	107.8	C22—C21—H21	120.3
C4—C3—C8	119.2 (3)	C21—C22—C17	121.5 (2)
C4—C3—C2	124.7 (2)	C21—C22—H22	119.3
C8—C3—C2	116.0 (2)	C17—C22—H22	119.3
C5—C4—C3	120.1 (3)	O4—N23—O3	124.0 (3)
C5—C4—H4	120.0	O4—N23—C12	116.1 (3)
C3—C4—H4	120.0	O3—N23—C12	119.9 (2)
C6—C5—C4	120.3 (3)	N27—C24—C13	115.53 (19)
C6—C5—H5	119.8	N27—C24—C25	87.13 (16)
C4—C5—H5	119.8	C13—C24—C25	115.6 (2)
C5—C6—C7	120.3 (3)	N27—C24—H24	112.1
C5—C6—H6	119.8	C13—C24—H24	112.1
C7—C6—H6	119.8	C25—C24—H24	112.1
C6—C7—C8	119.8 (3)	C28—C25—C26	120.7 (2)
C6—C7—H7	120.1	C28—C25—C24	120.22 (19)
C8—C7—H7	120.1	C26—C25—C24	84.93 (18)
C7—C8—C3	120.2 (2)	C28—C25—H25	109.6
C7—C8—C9	124.2 (2)	C26—C25—H25	109.6
C3—C8—C9	115.6 (2)	C24—C25—H25	109.6
C8—C9—C10	112.61 (18)	O5—C26—N27	131.2 (3)
C8—C9—H9A	109.1	O5—C26—C25	135.7 (2)
C10—C9—H9A	109.1	N27—C26—C25	93.04 (19)
C8—C9—H9B	109.1	C26—N27—C34	132.3 (2)
C10—C9—H9B	109.1	C26—N27—C24	94.87 (18)
H9A—C9—H9B	107.8	C34—N27—C24	129.76 (19)
N1—C10—C14	111.3 (2)	C33—C28—C29	117.6 (3)
N1—C10—C9	112.56 (18)	C33—C28—C25	120.3 (2)
C14—C10—C9	103.48 (18)	C29—C28—C25	122.2 (2)
N1—C10—C11	105.68 (17)	C30—C29—C28	120.8 (3)
C14—C10—C11	109.7 (2)	C30—C29—H29	119.6
C9—C10—C11	114.24 (19)	C28—C29—H29	119.6
C17—C11—C12	112.5 (2)	C31—C30—C29	120.6 (3)
C17—C11—C10	116.85 (18)	C31—C30—H30	119.7
C12—C11—C10	103.73 (18)	C29—C30—H30	119.7
C17—C11—H11	107.8	C30—C31—C32	119.4 (3)
C12—C11—H11	107.8	C30—C31—H31	120.3
C10—C11—H11	107.8	C32—C31—H31	120.3
N23—C12—C13	112.3 (2)	C31—C32—C33	120.9 (3)
N23—C12—C11	109.4 (2)	C31—C32—H32	119.6
C13—C12—C11	104.96 (18)	C33—C32—H32	119.6
N23—C12—H12	110.0	C32—C33—C28	120.7 (3)
C13—C12—H12	110.0	C32—C33—H33	119.7
C11—C12—H12	110.0	C28—C33—H33	119.7
N1—C13—C12	105.89 (17)	C35—C34—C39	119.0 (2)
N1—C13—C24	114.29 (19)	C35—C34—N27	120.5 (2)
C12—C13—C24	113.61 (18)	C39—C34—N27	120.5 (2)
N1—C13—H13	107.6	C34—C35—C36	120.5 (2)
C12—C13—H13	107.6	C34—C35—H35	119.7
C24—C13—H13	107.6	C36—C35—H35	119.7
O2—C14—O1	124.0 (3)	C37—C36—C35	120.2 (2)
O2—C14—C10	123.2 (3)	C37—C36—H36	119.9
O1—C14—C10	112.3 (2)	C35—C36—H36	119.9
C16—C15—O1	112.9 (3)	C36—C37—O6	125.5 (2)
C16—C15—H15A	109.0	C36—C37—C38	119.1 (2)
O1—C15—H15A	109.0	O6—C37—C38	115.4 (2)
C16—C15—H15B	109.0	C39—C38—C37	120.9 (2)
O1—C15—H15B	109.0	C39—C38—H38	119.6
H15A—C15—H15B	107.8	C37—C38—H38	119.6
C15—C16—H16A	109.5	C38—C39—C34	120.2 (2)
C15—C16—H16B	109.5	C38—C39—H39	119.9
H16A—C16—H16B	109.5	C34—C39—H39	119.9
C15—C16—H16C	109.5	O6—C40—H40A	109.5
H16A—C16—H16C	109.5	O6—C40—H40B	109.5
H16B—C16—H16C	109.5	H40A—C40—H40B	109.5
C22—C17—C18	117.5 (2)	O6—C40—H40C	109.5
C22—C17—C11	123.6 (2)	H40A—C40—H40C	109.5
C18—C17—C11	119.0 (2)	H40B—C40—H40C	109.5
C19—C18—C17	121.4 (3)		
			
C13—N1—C2—C3	82.0 (2)	C18—C19—C20—C21	0.9 (4)
C10—N1—C2—C3	−46.9 (3)	C18—C19—C20—Cl1	179.67 (19)
N1—C2—C3—C4	−131.6 (3)	C19—C20—C21—C22	−0.9 (4)
N1—C2—C3—C8	48.9 (3)	Cl1—C20—C21—C22	−179.73 (19)
C8—C3—C4—C5	0.5 (4)	C20—C21—C22—C17	−0.1 (4)
C2—C3—C4—C5	−179.0 (3)	C18—C17—C22—C21	1.1 (3)
C3—C4—C5—C6	1.2 (5)	C11—C17—C22—C21	−180.0 (2)
C4—C5—C6—C7	−1.8 (5)	C13—C12—N23—O4	−115.0 (3)
C5—C6—C7—C8	0.6 (4)	C11—C12—N23—O4	128.8 (3)
C6—C7—C8—C3	1.1 (4)	C13—C12—N23—O3	63.0 (3)
C6—C7—C8—C9	−179.4 (2)	C11—C12—N23—O3	−53.2 (3)
C4—C3—C8—C7	−1.6 (3)	N1—C13—C24—N27	−56.2 (3)
C2—C3—C8—C7	177.9 (2)	C12—C13—C24—N27	−177.9 (2)
C4—C3—C8—C9	178.8 (2)	N1—C13—C24—C25	−155.91 (19)
C2—C3—C8—C9	−1.6 (3)	C12—C13—C24—C25	82.4 (3)
C7—C8—C9—C10	135.8 (2)	N27—C24—C25—C28	−123.9 (2)
C3—C8—C9—C10	−44.7 (3)	C13—C24—C25—C28	−6.9 (3)
C13—N1—C10—C14	115.2 (2)	N27—C24—C25—C26	−1.17 (17)
C2—N1—C10—C14	−114.6 (2)	C13—C24—C25—C26	115.9 (2)
C13—N1—C10—C9	−129.2 (2)	C28—C25—C26—O5	−58.7 (4)
C2—N1—C10—C9	1.0 (3)	C24—C25—C26—O5	179.0 (3)
C13—N1—C10—C11	−3.9 (2)	C28—C25—C26—N27	123.6 (2)
C2—N1—C10—C11	126.3 (2)	C24—C25—C26—N27	1.27 (19)
C8—C9—C10—N1	44.5 (3)	O5—C26—N27—C34	−18.2 (5)
C8—C9—C10—C14	164.7 (2)	C25—C26—N27—C34	159.6 (3)
C8—C9—C10—C11	−76.0 (2)	O5—C26—N27—C24	−179.2 (3)
N1—C10—C11—C17	−103.8 (2)	C25—C26—N27—C24	−1.4 (2)
C14—C10—C11—C17	136.2 (2)	C13—C24—N27—C26	−115.8 (2)
C9—C10—C11—C17	20.5 (3)	C25—C24—N27—C26	1.31 (19)
N1—C10—C11—C12	20.6 (2)	C13—C24—N27—C34	82.5 (3)
C14—C10—C11—C12	−99.4 (2)	C25—C24—N27—C34	−160.4 (2)
C9—C10—C11—C12	144.93 (19)	C26—C25—C28—C33	156.5 (2)
C17—C11—C12—N23	−141.4 (2)	C24—C25—C28—C33	−100.4 (3)
C10—C11—C12—N23	91.4 (2)	C26—C25—C28—C29	−25.0 (3)
C17—C11—C12—C13	97.9 (2)	C24—C25—C28—C29	78.0 (3)
C10—C11—C12—C13	−29.3 (2)	C33—C28—C29—C30	2.3 (4)
C10—N1—C13—C12	−14.7 (2)	C25—C28—C29—C30	−176.2 (2)
C2—N1—C13—C12	−145.46 (19)	C28—C29—C30—C31	−1.1 (5)
C10—N1—C13—C24	−140.51 (19)	C29—C30—C31—C32	−0.3 (5)
C2—N1—C13—C24	88.7 (2)	C30—C31—C32—C33	0.4 (5)
N23—C12—C13—N1	−91.3 (2)	C31—C32—C33—C28	0.9 (4)
C11—C12—C13—N1	27.5 (2)	C29—C28—C33—C32	−2.2 (4)
N23—C12—C13—C24	35.0 (3)	C25—C28—C33—C32	176.3 (2)
C11—C12—C13—C24	153.8 (2)	C26—N27—C34—C35	29.3 (4)
C15—O1—C14—O2	2.1 (4)	C24—N27—C34—C35	−175.7 (2)
C15—O1—C14—C10	175.0 (3)	C26—N27—C34—C39	−151.7 (3)
N1—C10—C14—O2	−159.7 (2)	C24—N27—C34—C39	3.3 (4)
C9—C10—C14—O2	79.2 (3)	C39—C34—C35—C36	0.7 (4)
C11—C10—C14—O2	−43.1 (3)	N27—C34—C35—C36	179.7 (3)
N1—C10—C14—O1	27.3 (3)	C34—C35—C36—C37	−0.6 (5)
C9—C10—C14—O1	−93.8 (2)	C35—C36—C37—O6	−179.9 (3)
C11—C10—C14—O1	143.9 (2)	C35—C36—C37—C38	0.3 (4)
C14—O1—C15—C16	103.2 (5)	C40—O6—C37—C36	3.4 (4)
C12—C11—C17—C22	−30.5 (3)	C40—O6—C37—C38	−176.7 (2)
C10—C11—C17—C22	89.3 (3)	C36—C37—C38—C39	−0.2 (4)
C12—C11—C17—C18	148.4 (2)	O6—C37—C38—C39	179.9 (2)
C10—C11—C17—C18	−91.8 (2)	C37—C38—C39—C34	0.4 (4)
C22—C17—C18—C19	−1.2 (3)	C35—C34—C39—C38	−0.6 (4)
C11—C17—C18—C19	179.8 (2)	N27—C34—C39—C38	−179.6 (2)
C17—C18—C19—C20	0.2 (4)		

### Hydrogen-bond geometry (Å, °)

**Table:**

*D*—H···*A*	*D*—H	H···*A*	*D*···*A*	*D*—H···*A*
C2—H2A···N27	0.97	2.53	3.196 (3)	126
C24—H24···O3	0.98	2.58	3.186 (3)	121
C25—H25···O6^i^	0.98	2.60	3.479 (3)	150
C35—H35···O5	0.93	2.60	3.157 (3)	119
C40—H40A···O4^i^	0.96	2.49	3.232 (4)	134

Symmetry codes: (i) −*x*, −*y*+1, −*z*.
